# Wrist-Worn devices to encourage affected upper limb movement in unilateral cerebral palsy: Participatory design workshops

**DOI:** 10.3389/fresc.2022.1021760

**Published:** 2022-12-21

**Authors:** Rebekah Brown, Janice Elizabeth Pearse, Tom Nappey, Dan Jackson, Grace Edmonds, Yu Guan, Anna Purna Basu

**Affiliations:** ^1^ School of Psychology, Newcastle University, Newcastle upon Tyne, United Kingdom; ^2^ Population Health Sciences Institute, Newcastle University, Newcastle upon Tyne, United Kingdom; ^3^ Therapy Services, Newcastle Upon Tyne Hospitals NHS Foundation Trust, Newcastle upon Tyne, United Kingdom; ^4^ National Innovation Centre for Ageing, The Catalyst, 3 Science Square, Newcastle Helix, Newcastle upon Tyne, United Kingdom; ^5^ School of Computing Science, Newcastle University, Newcastle upon Tyne, United Kingdom; ^6^ Department of Biology, Northumbria University, Newcastle upon Tyne, United Kingdom; ^7^ Paediatric Neurology, Great North Childrens Hospital, Newcastle upon Tyne, United Kingdom

**Keywords:** hemiplegia, child, wearable device, upper limb, participatory design, MHealth (mobile health)

## Abstract

**Background:**

Unilateral (Hemiplegic) cerebral palsy (UCP) causes weakness and stiffness affecting one sided of the body, often impacting activities of daily living. Upper limb therapy at effective intensity is not accessible to most.

**Aim:**

To determine stakeholder views on design of an approach using wrist-worn devices and a smartphone application to encourage use of the affected upper limb for children with hemiplegia.

**Method:**

Four participatory design workshops and one young people's advisory group workshop incorporating views of five young people with hemiplegia, 13 typically developing peers aged 8–18 years, four parents, three occupational therapists, one teacher and two paediatricians. Two special educational needs co-ordinators were consulted separately. Peers were included to explore a study design whereby each child with hemiplegia would have a participating “buddy”. Topics included views on an acceptable wrist-worn device and smartphone application, participant age range, involvement of a buddy, and barriers to using the technology in a school setting. Ethical/welfare considerations included data security, and potential risks around providing smartphones to young children.

**Results:**

Children wanted a comfortable, conventional-appearing wristband incorporating a watch face and a secure, well-fitting strap. They were prepared to wear a band on each wrist. They wanted support with explaining the study to schoolteachers. Most schools restricted smartphone use during the school day: the study design accommodated this. Children agreed with a game as reward but had different views on an acceptable game; direct access to feedback data was preferred by some. Parents commented on the lack of access to upper limb therapy for children with UCP; therapists concurred. The proposed participant age range was widened based on feedback. Typically developing children were prepared to be buddies to help a friend with CP. Stakeholders were reassured by data security explanations and plans to provide internet safety information to participants.

**Conclusion:**

The participatory design process informed plans for the proof-of-concept stage of the study, hopefully leading to an approach that will be fun, easy to integrate into everyday life, and have the capacity to increase use of the affected arm and hand.

## 1. Introduction

Unilateral (hemiplegic) cerebral palsy (UCP) affects around 39,000 people in the United Kingdom. UCP causes weakness and stiffness of one side of the body, impacting on activities of daily living (1, 2), peer relationships (3), quality of life and self-esteem (4), with long-term consequences for independent living and employment (5, 6). Intensive therapy interventions improve upper limb function in children with UCP compared with usual (low-frequency, low-intensity) care (7). Intensive interventions require high levels of resources (e.g., 1:1 staffing levels, 8 h/day in 2-week blocks) and are unavailable to many children. Approaches such as constraint therapy (in which the less-affected arm is restrained) are somewhat intrusive, and still require intensive therapy to promote use of the affected arm (8–10). Modifications of intensive therapy for home-based delivery are being explored (11). Fitting therapy around the home and school routine is challenging and requires motivation, patience and understanding: children with UCP require more practice than their typically developing peers to learn motor tasks (12). It is hard to achieve adequate practice throughout the day without “nagging”. There is a need for an acceptable solution, that motivates and supports children with UCP to independently increase use of their affected upper limb in a real-world setting, without intensive therapist or parental input. One option to consider is wearable technology.

Use of wearable technology to monitor activity levels (13) has the potential for marked behaviour change (14, 15). Smartphone applications and other “mHealth” technologies (mobile technologies for health) (16) promoting self-monitoring and self-management are becoming popular for young people with chronic health problems, e.g., diabetes and epilepsy (17, 18). There are significant challenges in developing mHealth interventions tailored to children (19). Nonetheless, we considered that a well-designed mHealth approach could be valuable for children and young people with UCP, by monitoring and providing feedback on arm activity. A similar approach was recently piloted in adults with hemiplegia following stroke at our institution. The device contained an accelerometer to monitor arm activity; it also reminded them to use the affected arm by vibrating discreetly if activity during the previous hour fell below a personalised, agreed threshold. In a pilot trial of this system, there was a 16% increase in arm activity (counts per minute) in the hour after a prompt, compared with the preceding hour. A pilot randomised trial (in which both groups received a therapy program and wristbands but only the intervention group received prompts to increase limb movement *via* the wristband) provided further positive feasibility data regarding the approach (20). We considered whether a similar approach could be used for children, with appropriate modifications to account for age-related lifestyle differences (e.g., school).

Children's perceptions of activity monitoring devices have been explored in other settings including the context of cystic fibrosis (21). Wrist-worn devices and devices providing feedback were particularly acceptable. A study of pre-adolescent school-age children found that comfortable, well-fitting, waterproof wrist-worn devices with engaging features were associated with the greatest compliance (22). Use of accelerometry is thus a promising real-world solution for children with UCP, empowering them to take control of their own improvements using technology with which they are familiar and comfortable. However, children's activity levels vary greatly over time – a system is needed that compares current activity with activity on the most recent day with similar structure (e.g., school day vs. weekend), so performance can be monitored through the school term. Another way to assess relative progress in arm activity of the affected side in UCP is to compare it with activity of the unaffected arm, but this requires the participant to wear two wristbands.

Use of wrist-worn accelerometers to assess arm movement in children aged 3–16 years with UCP has recently been validated by comparing counts with video-annotated movement episodes (23), comparing the movements of the affected and unaffected arms, and comparing movements of children with hemiplegia and typically developing controls, whilst undertaking an assessment of upper limb function (24). Feasibility of their use to monitor changes in arm activity after constraint-induced movement therapy has also been demonstrated (25). In these studies, children wore accelerometers on both wrists. This has the advantage of being able to calculate the ratio of movement of the affected arm to the unaffected arm, thus controlling for more general body movements to an extent. The arm movement ratio was shown to correlate strongly with the Fugl-Meyer Motor Assessment (a disease-specific index of arm motor impairment) in adults with subacute stroke (26). However, these studies did not involve longer-term commitments to wearing of devices on both wrists.

To our knowledge, no previous study has combined the concepts of using collecting real-world movement data from both wrists over a prolonged period in children with hemiplegia, providing prompts to move and feedback based on relative arm movements through a smartphone application. We intended to pilot this approach to see if it had the potential to increase activity of the affected upper limb without requiring intensive therapist input. We also intended to include data from a “buddy” or typically developing peer in the pilot study. Buddy systems have been shown to be valuable in other settings – for example, physical activity levels of children can be positively influenced by the activity of a “buddy” or “best friend” (27). There were several reasons for the planned inclusion of a buddy for each participant with UCP in the future pilot study. Firstly, contemporaneous information on relative arm use in typically developing peers in the same setting as the child with UCP could help with data interpretation (for example if both children were attending the same classes at the same school). Secondly, having a buddy system provides a mechanism for inclusiveness and mutual support, may prevent the child with UCP from feeling “singled out” (28).

Our overarching aim was thus to develop and pilot a software interface for a wrist-worn device, and a smartphone application for children with UCP, providing performance-related feedback to reinforce and reward activity of the affected upper limb in everyday life. This approach could reduce reliance on therapist input, allowing more efficient use of the limited therapist contact time available. This paper reports the user-centred design phase prior to the pilot study. Given the innovative nature of the planned pilot study, the user-centred design phase was considered critical to optimise the components of that study. In this phase, we undertook workshops with stakeholders to consider the features required in the wristband and enhance development of the smartphone application through prototype evaluation, to guide iterative design change. We also obtained feedback to optimize the design of the pilot study, including the involvement of buddies.

## 2. Materials and methods

A user-centred design approach was followed, in which refinement of the concept occurred through a series of four mixed-group workshops with key stakeholders, as well as one workshop with the Young Person's Advisory Group North England (YPAGNE). Email and telephone correspondence was used to obtain views from stakeholders who were unable to attend workshops. This phase of the research fell into the Stakeholder Involvement and Establishing a Team for Decision-making process stages in the MRC Intervention Development guidance (29).

### 2.1. Research team and reflexivity

Four members of the research team (AB, DJ, TN, JP) were actively involved in facilitating the first four workshops due to the broad range of issues to be covered. AB independently facilitated the final group with YPAGNE. AB is a female consultant paediatric child neurologist and clinical senior lecturer with credentials BMBCH MA FRCPCH PHD, and experience in mixed methods research projects in the field of childhood neurodisability. DJ is a male senior research associate with a MEng computer systems and software engineering degree, and experience in ubiquitous computing and technologies to support health and wellbeing including projects involving participants with stroke. TN is a male senior user experience designer with credentials BA in Design for Industry and a background in the field of industrial design. JP is a female senior paediatric occupational therapist with credentials DipCOT MPhil, with clinical and research experience in upper limb assessment and rehabilitation in children with neurodisability. Due to the nature of the research, most participants who were not typically developing children and were already known to AB and/or JP from a clinical context. The reasons why the team were pursuing this line of research were made clear in the information sheet and reiterated during the workshop introductions.

### 2.2. Participants, recruitment and setting

Sampling was purposive in that we wished to obtain feedback from children with UCP and their parents, typically developing children, occupational therapists, paediatricians, teachers and special educational needs co-ordinators (SENCOs). Recruitment was through social media, including through local patient support groups with help from our parent co-applicant. Ethical approval was obtained through the Faculty of Medical Sciences Ethics Committee, Newcastle University.

We initially recruited children aged 12–15 years but then successfully sought approval to extend the age range from 8 to 18 years. The increase in the upper age limit was due to expressed interest from a 16-year-old potential participant; the lower age limit was decreased due to feedback from the workshops that the proposed technology might be appealing to and appropriate for children who were younger than we had originally envisaged, as discussed in the Results section below. We also successfully sought an amendment to approach SENCOs in the region *via* a letter sent out through the head SENCO acting as gatekeeper, and to follow up remotely, as no SENCOs attended the workshops.

Written informed consent (or assent for children) was required from all participants prior to taking part, with the exception of YPAGNE. YPAGNE is an advisory group of children and young people supporting clinical research studies in children, including the planning phase (About | Generation R). Workshops were held in meeting rooms in a university building in central Newcastle; the YPAGNE event was held in a clinical learning environment within the grounds of Newcastle upon Tyne Hospitals NHS Foundation Trust. Summary data only regarding YPAGNE attendees was provided, owing to the nature and role of the group.

### 2.3. Procedure

Each workshop included an introductory presentation about the project rationale and aims. A topic guide (Supplementary Appendix) was agreed upon by all workshop facilitators and shaped the subsequent content of the workshops whilst allowing scope for free discussion.

Several commercially available wrist-worn devices were brought to workshops for participants to try on, leading to discussions regarding their comfort, size, shape and appeal as well as technical considerations such as how to charge the devices. We explained that we anticipated that the young people in the study would be wearing a wristband on each wrist all day each day for 10 weeks; that each band would record information regarding arm movements; that this information would be sent to a smartphone application and compared with baseline data; and that for participants with hemiplegia, a reminder to move their affected arm would be given if movement fell below a pre-set agreed threshold. We asked for views on the feasibility and acceptability of this approach, and any perceived pitfalls.

Participant wishes for the design of an associated smartphone application providing further feedback and motivation were also discussed. In relation to this, we explored the potential for including a game as an “incentive” to continue to participate, and considered what types of game might work well, and why. We also asked what kind of feedback on performance the young people would find helpful and in what format.

We explained that, for a mixture of reasons including “moral support” and to provide additional feasibility data, we hoped that each young person with hemiplegia would choose a “buddy” of similar age who did not have hemiplegia but who would also take part in the study. We asked workshop participants for their views on this including any reservations. Finally, we also covered practicalities around school, and any potential pitfalls anticipated in a future proof-of-concept study. The YPAGNE group session focused on what would motivate a “buddy” to take part and whether they would be prepared to wear two wristbands for the duration of the study.

SENCOs from schools in the region were unable to attend the workshops but were contacted by email through the regional SENCO lead, with information about the project and a list of remaining questions. These questions covered whether they had any reservations about the wristbands being worn at school; if there were activities for which wristbands should not be worn; school policy on fitness trackers and smartphones; and any other issues they wanted to raise.

Workshop sessions were audio recorded, then transcribed verbatim and pseudonymised. For the YPAGNE group, contemporaneous notes were taken and subsequently summarised. A team debrief took place after each workshop apart from the final YPAGNE session, field notes were collated, and any issues highlighted and discussed to inform subsequent workshops. By the end of the fifth workshop, it was felt that no new issues were arising,.

### 2.4. Data analysis

Thematic analysis was undertaken according to standard procedures (30). We used procedures from first-generation grounded theory (coding, constant comparison, memoing) (31) and from constructionist grounded theory (mapping) (32). More specifically, we used a form of content analysis known as the Framework approach (33, 34) which is particularly suited to applied qualitative research (35). First, RB gained familiarity with the transcripts; then, coding was undertaken with cross-checking by AB. The research team jointly discussed the interpretation of key issues emerging from the data. An analytical framework was developed based on review of transcripts (Table 1) which was used to index the transcripts and chart the data into the framework matrix using Microsoft Word. Notes were made referencing key illustrative quotations. Using the matrix, common themes and subthemes could be identified and described. We did not ask participants to provide feedback on the overall findings.

**Table 1:** Coding tree.

**Appearance**	***Device***	How does the device look?
				Is it attractive?
				Is it discreet?
				Is it an appropriate size and shape?
				Is wearing it going to look out of place?
				How do you tell the difference between left and right?
				Will there be a screen? A clock?
		***Self***	Views on wearing two devices?
				Self-conscious?
**Ease/Practicality**	***Device***	Is device simple/easy to use?
				Does its use fit into real life?
				Does it adversely affect day to day activities?
		***Strap function***	Is it easy to put on?
				Is it comfortable?
				Does it stay on?
		***School life***	Will teachers allow wristband?
				Explanation of involvement in the study?
				Can they keep wristband on during physical education classes?
				Can they have their phones in school?
**Incentives**		What will motivate them to take part? (Child with unilateral cerebral palsy; Buddy)
				What will make them want to move their arm more?
				Game: Would a game motivate them? Types of game
				Would seeing their data on the smartphone application motivate them?
				Lack of therapy: inability to get help elsewhere
**Ethical/Welfare issues**		Access to arm movement data: General Practitioners? Parents?
				What can be shared with the Buddy?
				How detailed is the movement data collected?
				Internet safety
**Protocol**		Age group
				Duration of study
				Buddy

## 3. Results

Five children and young people with UCP (age range 8–16 years; 4 male), four of their parents (all mothers), one typically developing 13-year-old boy, three occupational therapists, two paediatricians (one neurodisability consultant and one trainee) and a retired teacher (all female) took part in the first 4 workshops. Apart from the TD child (who attended three workshops), all other invitees each attended one event only. All workshops involved representation from more than one stakeholder group. A further two occupational therapists, plus one boy with hemiplegia and his mother had agreed to take part but were then unable to attend, due to work commitments and health issues. GE (female undergraduate student) and YG (lecturer in computing science and co-applicant) assisted with workshop preparation and were present at the first two workshops, The first four workshops were each around 90 min in duration. No SENCOs attended the workshops but email responses to targeted questions were obtained from two SENCOs. The YPAGNE workshop was attended by 12 young people (2 male), age range 15–18 years, mean age 16.5 years and lasted 45 min. Thus, in total the views of 30 stakeholders were obtained.

Themes emerging from the data were: Appearance (of wristbands), Ease/practicality of use (in relation to wristbands and smartphone application, and particularly in a school setting), Incentives/motivation to participate, Ethical/welfare issues, and Suggestions regarding the protocol (Table 1).

### 3.1. Appearance of wristbands

The appearance of the wrist-worn devices was an important issue for the young people. A conservative colour and appearance (including the presence of a watch face, as would be seen on most other wrist-worn fitness trackers) were favoured and were incorporated into the prototype.

“Can it not just have a watch face or something instead?” – [03, boy with hemiplegia, age 13, workshop 1].

Another aspect relating to appearance was the need to have a device on each wrist. None of the participants said that the need to wear two wristbands would put them off taking part. One participant suggested that one device could be kept “under your sleeve” [01, boy aged 13, workshop 1], suggesting an attempt at appearing to wear only one device. A parent of a younger child said that it might even be a useful talking point:

“I think [name] would be quite happy to wear the two because he”s desperate for a one anyway, but having two would have a novelty value for him, and he”d be able to tell people why, and he quite enjoys, at this minute, at this age, telling people why he”s doing things” *[20; Mother of 8-year old boy with hemiplegia, workshop 4]*.

### 3.2. Ease/practicality

Simplicity of use was important to participants. This included practicalities around the design and function of the strap and device, and practicalities of use during the school day.

#### 3.2.1. Strap and device

Participants with hemiplegia struggled to put the wrist-worn devices on by themselves; one participant described the process as “fiddly” [03, boy with hemiplegia, age 13, workshop 1]. There was some discussion about whether bespoke Velcro straps would be easier to use [04, occupational therapist, workshop 1], though the research team had avoided that solution due to the lack of aesthetic appeal compared with commercial straps. Instead, various commercial strap designs were tested for ease of use. It was acknowledged that some participants would need to ask for help with fastening and unfastening the straps especially when attempting to do so with the affected hand [04, occupational therapist, workshop 1] but it was felt that this help would generally be readily available:

“Your TA (teaching assistant) would probably do that for you”: [17; mother of 8-year-old boy with hemiplegia; workshop 4].

“…or he”d just ask his buddy” [20; mother of 8-year-old boy with hemiplegia, workshop 4].

The challenges of supinating the affected forearm (so that the underside of the wrist faced upwards to fasten or unfasten a strap) were also acknowledged [04, occupational therapist, workshop 1]. Discussions were had in workshop 1 regarding the benefits and disadvantages of different types of fastening, with no clear conclusions. A balance had to be sought between ease of securing the bands and likelihood that they would come loose and be lost. A challenging factor was the relatively small wrist size of child participants, particularly regarding the side affected by hemiplegia [02, mother of 13-year-old boy with hemiplegia, workshop 1], which was smaller by 2 cm in one participant than the dominant side.

“I”ve never worn a watch, as they”re too big for me” [10, girl with hemiplegia, age 16, workshop 2]

Whilst the straps chosen were able to accommodate the small wrist sizes, the size and rigidity of the rectangular “watch face” component containing the circuitry was identified as a potential problem for those with the smallest wrists. An alternative shape and smaller size of this component might produce a better fit on small wrists, and allow for a thinner wristband, but the project was constrained by the need to use currently available devices. Participants would also have preferred a waterproof device, but this was not possible given the requirement to remove the “watch face component” for charging *via* a USB port:

“What, take them off every time I go to the shower?” [01, boy aged 13, workshop 1]

The final choice of device and wristband accommodated both a secure fastening design and a reasonably comfortable silicone strap, which participants were happy to wear for the duration of the workshops; though one participant [19, 8-year-old boy with hemiplegia] felt that “it got hot” under the strap, during a workshop held on a particularly hot summer's day.

“I think the newer band is better. It”s more comfortable and it”s less fiddly”. [01, boy aged 13, workshop 2]

“It”s definitely more secure isn”t it!” [11, occupational therapist, workshop 2]

It was important to find a simple way for participants to distinguish between the left and right wrist-worn devices and straps, so that they could be placed consistently on the correct wrists. This issue was raised in workshops 1, 2 and 3, e.g.,:

“How can you tell which is right or left?” [02, mother of 13-year-old boy with hemiplegia, workshop 1]

“How would you know if he took it off this arm and put it back on the right one if it”s not marked?” [12, mother of 15-year-old boy with hemiplegia, workshop 2].

By workshop 3, the research team proposed using a toucan icon (as a reflection of the project name “TwoCan”) appearing on each screen display, so that the toucans would be facing each other when the devices were on the correct wrists. For typically developing children a picture of a parrot was used, to make it easier to distinguish between devices belonging to a child with UCP and their buddy. A prototype demonstrating this solution was shown to participants at workshop 4. The practical importance of this was to prevent the child with UCP and buddy from accidentally wearing each other's bands. The use of a screen display rather than an external marker increased the flexibility of reallocation of the bands between participants.

#### 3.2.2. School life

There was some initial trepidation at the idea of having to wear the devices at school. This was particularly in the context of the device emitting a “beep” or vibrating as a prompt to increase movement of the affected arm during a lesson:

“I”ll get told off” [03, boy with right hemiplegia, aged 13, workshop 1]

Exploration of this led to parental elaboration that teachers would assume the sound came from a mobile phone [02, mother of 03, workshop 1], and phones were not permitted during the school day. Of the two SENCOs responding, one [participant 22] concurred as their school prohibited mobile phone use; this SENCO was concerned about whether the noise emitted by the device would distract other students; the research team replied with reassurance on this point the sounds are brief, infrequent (occurring hourly at most) and quiet. The other SENCO [participant 21] stated that phone use was only banned during examinations. Importantly for the project, daytime prompts from the device are not reliant on proximity to a mobile phone. Synchronisation between devices is necessary for the participants to be able to review their progress on the smartphone application but could be done after school. Even though the proposed project would not require the children to access a smartphone during the school day, it was clear that the children needed a simple mechanism to alert their teacher to their participation in the study in the event of questions. A “business card” was designed for this purpose. We also explored the option of using a different form of prompt such as a flashing light, but this option was quickly dismissed because of the likelihood that children would hide the devices under their sleeves [03, 13-year-old boy with hemiplegia, workshop 1]. Children with UCP confirmed during the workshops that they could sense the vibratory prompts emitted by the devices.

Some schools also have a policy against the use of activity monitors during school hours. Of the two SENCOs, one [participant 22] stated that fitness trackers were allowed in school and the other [participant 21] stated that they weren't. This might be problematic even if special permission were to be provided for participants in this project:

“It might create conflict, because some people might start going “well I want to wear *my* Fitbit”.” [11, Occupational therapist, workshop 2]

Also relating to school life was use of the devices during physical education (PE):

“In sport we are not allowed to wear watches” [01, TD boy aged 13 years, Workshop 2]

Both SENCOs were prepared to support the wearing of the wristbands for the project, except for PE depending on the specific activity being undertaken [22] or the view of the PE teacher [21].

### 3.3. Incentives/motivation

Another important theme that emerged from the data was that of incentives. This can be conceptualised as factors which would encourage participation in the project, and factors which would support those with UCP to move their affected arm more. Four sub-themes were identified: lack of alternative options; locus of control; a game as reward, and feedback of summary data. One potential disincentive was a concern about whether the level of detail captured by the arm movements constituted a potential privacy breach – for example, whether it would be possible to pick up private information such as PIN numbers from arm movement. The group was reassured on this point, as the stored data are averaged over an epoch of one minute.

#### 3.3.1. Lack of alternative options

Some parents of children with UCP attending the workshops identified problems with their child's current level of access to therapy, and with waiting times:

“The physio, I”ve found, just dwindles off (with age)” [02, mother of 13-year-old boy with hemiplegia, workshop 1]

Therapists expressed similar concerns and explained that their service was commissioned only to provide short duration therapy interventions [05, occupational therapist, workshop 1]. Lack of alternative options was a driver for consideration of alternatives such as participation in this study.

#### 3.3.2. Locus of control

One mother expressed relief that approaches shifting the locus of control to the young person were being explored:

“I love the idea that they are in control of their own physio almost, you know, because it takes the pressure off us as I am not having to nag” [17, mother of 8-year-old boy with hemiplegia, workshop 4].

#### 3.3.3. Game

To motivate and reward participants for engaging with the study, incorporation of a game into the smartphone application was suggested by one of the facilitators (TN). Points could be accrued for good progress (or for TD participants, for ongoing engagement). This was a clear incentive in some cases:

“By moving your hands you”d get points - and points mean you get to play a game at the end of the day on either Mum or Dad”s phone” [17; Mother of boy with hemiplegia, workshop 4]

“Yeah, yeah, yeah!!!” [Son; 8 years old, left hemiplegia]

Challenges were quickly identified, in that participants varied greatly regarding whether a game would interest them (and for how long), and if so, what type of game. Some children were interested in a multi-player game: the competitive element was appealing.

“The more active you get, the further along a scale that you get, and you can compete with your friends” [01, Boy, 13 years, workshop 1]

However, this would require moderation with respect to each young person's performance capacity.

There were further ideas about what, specifically, the “reward” within the game could be:

*“*You update like, your character” [03; Boy with hemiplegia, 13, workshop 1]

“If the feedback could be made into some sort of challenge, then I would be interested” [01; Boy, *13,* workshop 2]

There was a long discussion in both workshops 1 and 2 about the types of games played by schoolchildren on their smartphones, but no solution presented itself that would appeal to all.

#### 3.3.4. Summary data

Some participants were not interested in having a game, and just wanted to see an accessible version of the processed data as feedback: when asked “if you could get anything from wearing that band, what would it be?”, a 16-year-old with hemiplegia [10; workshop 2] replied, “Probably the data. I like research so I would like to look at the data”. She added: “You could sort of have an app where it just shows what you”ve done”.

The opportunity to see live accelerometry data during the workshops motivated participants to think about their relative use of each arm:

“It is mostly just my leg we pay attention to” [19; Boy, 8 years old, right hemiplegia, workshop 4]

“It is! And I have never really focussed on how much he does or does not use his arm, and what he does use it for…” [20; Mother of 19]

“Did your arm go up when you were running?” [17: Mother of child with hemiplegia, workshop 4]

“Err, yeh! And sometimes when I am running it makes it easier if I go like this with my arms” (demonstrates large circling arm movements) [19]

One participant expressed a hope that other atypical movements could be detected using the wristbands and shared with other healthcare professionals such as the general practitioner:

“What about kids with tremor and fitting…And funny movements that shouldn”t be there” [12; mother of 15-year-old boy with hemiplegia, workshop 3]

Similarly, a neurodisability consultant wondered whether the data could be used in clinic [participant 15, workshop 3]. Ethical, interpretational and practical issues limit these potential uses at present but could be considered in the future.

#### 3.3.5. Motivation for the buddy

One participant asked what would motivate a buddy to take part in the project [16, Teacher, workshop 3]. Based on feedback from the YPAGNE advisory group, altruism would pay a part in this. Typically developing children attending the YPAGNE session were asked if they would be prepared to take part as buddies. One answered that they would do so for a friend: paraphrasing, though they might feel slightly under pressure “because it”s a friend, in the end all you would have to do is wear two wristbands and look at an app”. It was felt that it would be better for a friend to take part than to have a volunteer from the class as buddy. When asked whether they would be prepared to wear two wristbands, one replied that it was “slightly more annoying” to have to do this than to wear only one band, but that they would do so.

### 3.4. Ethical/welfare issues

#### 3.4.1. Sharing of data

Participants varied in their comfort with sharing their data remotely with their therapists:

“The thing is with therapists knowing data they will then know what to do next” [10; 16-year-old girl with hemiplegia; workshop 2]

“I can see how he would think “I have got to do it because people will know if I haven”t”, and that will become like when I tell him he has got to do his exercises and he does not want to.” [20, mother of 8-year-old boy with hemiplegia, workshop 4].

A member of the YPAGNE advisory group asked about whether provision of movement data constituted a potential security issue, for example whether if a participant was typing their PIN (bank security) number into a machine, this number could be discerned. They were reassured on this point, in that the bands recorded data from the wrists not fingers, and then only stored data averaged over a minute at a time, so this level of detail would not be resolved.

#### 3.4.2. Access to smartphone

One parent was reluctant for her child to have access to a smartphone, because of the potential for unregulated access to the internet. The option of installing the application on to the child's “tablet” and thus controlling internet access was raised by this parent.

“So, it if was an app for [name], it would have to be on my phone, …but the issue for that would be that it has access to an awful lot of other things also”. [20, mother of 8-year-old boy with hemiplegia, workshop 4].

### 3.5. Suggestions for proof-of-concept study

Participants were generally in support of the proof-of-concept study including duration. A helpful suggestion to broaden the age group to include younger children was made:

“It”s just I have a few patients who are KS2 ages (7–11), and they would love it, and feel grown up doing it” [11, occupational therapist, workshop 2].

We therefore included two young (8-year-old) children with UCP in workshop 4. In contrast to the children in the previous workshops, neither owned a smartphone.

– “He”s desperate for a one anyway” [20, mother of 8-year-old boy with hemiplegia].
– “[His] sole purpose in life is to have access to a telephone” [20, mother of 8-year-old boy with hemiplegia]

Both children explored the technology with enthusiasm and one boy [participant 18] could be heard whispering plans to his mother about who could be his buddy in the proposed study.

There were some reservations about a buddy system given the possibility that a young person might “fall out” with their buddy within the 10-week study period, so flexibility within this system was encouraged:

“So, if it (relationship with buddy) did sort of fall apart, say five weeks in, would it be ok to change?” [20, mother of 8-year-old boy with hemiplegia].

## 4. Discussion

Our workshops were instrumental in optimising the design of the main TwoCan study, including the choice of wrist worn devices, details of firmware, and the design of the phone application as well as suggestions regarding the protocol. This is in line with our previous positive experience of participatory design processes (36) and with the broader literature. Involving the end-user at the design stage can lead to better generation of ideas for solutions that optimally meet user needs, therefore more successful innovations (37); it is also likely to improve user satisfaction with the final product (38).

### 4.1. Changes made following the workshop

Design principles for the devices, smartphone application and game, ethical concepts and protocol, shaped by the workshops, are summarised in Table 2. There was understandably much focus on the appearance, functionality and ease of use of the wristbands, as well as their comfort. This resonates with previous reports of the importance of comfort, support of interaction, scalability to body size diversity, and ease of use, for wearable devices (39). These design principles will help to optimise the equipment, firmware, software and protocol for the pilot study.

**Table 2:** Summary of design principles.

**Devices - practicality/ease/comfort/aesthetics**	Easy to fit
	Wearing two wristbands was acceptable
		Appear to be a standard fitness device (e.g., show time)
		Ability to distinguish left/right
		Longevity of battery and simplicity to charge
		Privacy/security of the device (summarize data into one-minute epochs, locked access)
		Synchronize data when possible (does not need mobile device continuously connected)
**Smartphone application and game - incentives**	Simple/fun game
		Synchronize data from bands and to server so that “points” can be earned towards game and that data can be viewed on application
		See own data
		Game as an incentive (cooperative but with a competitive edge; smartphone-friendly; longevity)
		Privacy (device locked, paired to device, access token can be revoked)
**Protocol for proof-of-concept study**	Participant age group broadened
		Buddy is optional
		Information card for school; ensure teachers are aware
**Ethics and participant wellbeing**	Privacy/security of the device (summarize data into one-minute epochs, locked access) – minimise what data is shared with the buddy
		Finding a buddy and not being left out.
		Young child having access to a smart phone (phone needs data access to sync – Internet safety education leaflet

#### 4.1.1. Protocol

One of the most challenging issues was to decide on the appropriate age range for children with UCP to be included in the study protocol. Our original proposal focussed on children aged 12 to 15 years. This was because young people aged 16–18 years often have other significant pressures such as preparation for external examinations, and those aged under 12 years are less likely to have a smartphone. Ofcom reported that in 2020, smartphone ownership by United Kingdom children was 14% for those aged 5–7 years, 49% for those aged 8–11 years and 91% for those aged 12–15 years (40). The workshop discussions indicated that younger children might be particularly keen to take part precisely because they did not already own a smartphone and would be excited to have access to one. We therefore widened our age group inclusion criteria to 8–18 years.

Parents of younger children were concerned about internet safety if their children had access to a phone and were relieved that the application could in theory be downloaded on to a tablet or separate “study phone” for which parental controls on internet access could be set. This is in line with the 2021 OFCOM report on media use and attitudes which states that 98% of parents in the United Kingdom moderated their children's online access in some way (40). We planned to include internet safety information for families in the proof-of-concept study.

This broadening of the age range inclusion criteria necessitated careful thought regarding the nature of any proposed feedback or incentivization through the smartphone application. Any game should be easy to use and fun across the age range and any summarised data should be as accessible as possible to all. In fact, discussions indicated that there was no real consensus regarding a game which would appeal to all, regardless of age considerations. We decide to include a game as optional and to provide simple summary data for all.

One of the challenges of including younger children in the study was the lack of wristbands of a size suitable for smaller wrists which would fit the study purpose. This was a particular problem for children with UCP, for whom the affected upper limb is often a little smaller than the unaffected one. Interestingly, none of the children with UCP in our study already owned a fitness tracker. There is increasing interest in using wrist-worn devices to obtain clinically useful data for children (41, 42); some commercial companies producing fitness trackers and smartwatches now also sell devices targeted at younger age groups, therefore future studies are unlikely to encounter this difficulty.

Severity of the hemiplegia is unlikely to be a major limiting factor for involvement in this study. It is likely based on the workshop findings that most participants with hemiplegia will need (and be able to access) some help with fastening and removing the wristbands; and that they will use their dominant hand to access the smartphone application. The approach may however be particularly appealing to young people wishing to focus on increased movement of the affected upper limb rather than on increasing manual dexterity (1).

### 4.2. Issues relating to the school environment

Schools differ on their policies regarding mobile phone and fitness tracker use; however, there was a sense from parents that the project could likely be accommodated in most cases if mobile phones were not required to be used during the school day. This was in line with discussions with the Senior Adviser for SEN and Special Schools for the region, and from feedback from SENCOs; and was feasible. As a result of feedback from the workshops, information sheets for schools were created for the proof-of-concept study, as well as small cards for children to hand out to explain their participation to teachers. We also sought help from the Senior Advisor for special educational needs and special schools, who agreed to circulate information about the study to local SENCOs.

### 4.3. Incentives/motivation

Gao et al. conducted a large survey in China exploring the factors that influence consumers' decisions about whether to use wearable technology in healthcare (43). A distinction was made between “fitness wearable devices” (e.g., monitoring step count) and “medical wearable devices” (to monitor conditions such as diabetes). Our system falls more into the “fitness wearable” category, but its use in children with hemiplegia merits examination of views from the latter category too. The findings from their study, matched to considerations from our study, are listed in Table 3. The main factor missing from Gao's list but identified in our study by parents and therapists was the lack of alternative options to support upper limb function in their children; the other factor specific to our study was the indication that altruism would motivate potential buddies.

**Table 3:** Predictors of use of wearables (adapted from Gao et al. 2015).

Factor: most significant predictors listed first	Definition	Relevance for our study
**Social influence**	Extent to which others’ perceptions influence the consumer's decision	Relates to issues about “fitting in” at school; wearing two bands mitigated by involving buddy? Acceptable design critical.
		**Perceived privacy risk**	Extent to which consumers believed their data was safely protected	Participant questions about data safety addressed.
**Effort expectancy (Medical wearables group)**	How easy to use (or otherwise) the consumer perceives the technology to be	Wearables and Application straightforward to use
**Self-efficacy (Medical wearables group)**	Capacity of the user to effectively use the wearable to self-monitor and self-manage	Key outcome of proof-of-concept study. Relevant to “Locus of control”
**Perceived vulnerability**	Possibility that the consumer will experience a health threat	Participants with hemiplegia are already affected but need to avoid deterioration in function as a minimum
**Perceived severity (Medical wearables group)**	Extent of the threat from unhealthy behaviours	Parents aware of the importance of ongoing maintenance of function in the absence of regular therapist input
**Hedonic motivation (Mostly fitness wearables group)**	Pleasure derived from using the technology	Motivation to continue; quality of application and game
**Functional congruence (Mostly fitness wearables group)**	Perceived value for money of the device for the specific end user	Not relevant as no cost to participants during study

It was important to establish, as we did, that participants were prepared to engage with our approach. There is one report of a prototype worn on both wrists in adults with stroke (44) – however, their design features made the devices difficult to use, Another preliminary study in adults with stroke (45) used vibrotactile cueing to the paretic arm very frequently (every 30 s during five minute tasks) whilst recording activity of each arm *via* accelerometers. This required the 5 participants to wear large wristbands on each arm, extending well up into the forearm. Whilst they reported acceptability of the approach, our workshops suggested that such frequent cueing and large devices might not be well tolerated in children.

One of the strategies that a smartphone application can exploit is the use of incentivisation and reward. In one study, use of a game as a reward and a way of providing feedback on progress was shown to be enjoyable for participants; however, the goal of the approach, which was to improve fitness, was not achieved (46). Participants in that study felt that their motivation would have been higher if the game had been more challenging. It will be important in the next phase of our study to ensure any feedback incorporated can be pitched at the right level of interest for the participants (e.g., clear representation of data; games at appropriate levels of difficulty). It will also be important to capture both subjective views and objective evidence of change in arm use in relation to use of the approach.

The game itself will need to be simple and fun, and achievable on a mobile phone by children with UCP. A simple “launcher” genre game lends itself to this approach. We intend to incorporate a reward system into the game. Within this system, participants can influence the number of points they receive during the day by the degree to which they comply with the request to wear the devices for the stipulated time each day, and (for children with UCP) by the relative movement of the affected arm with respect to the unaffected one. Points can then be used within the game to assist progression through it *via* accessing “upgrades”.

With time, the novelty of interventions such as activity trackers may wear off, though this is partly counterbalanced by an increase in ability of participants to use the devices more intelligently (e.g., adjusting their targets based on plans for activities during the day) (47). 63% of those who had owned a fitness tracker for 1–3 months did not change the way they used their device, i.e., they continued to look at the data regularly (48). It remains to be seen whether children will be able to sustain use of the devices over a 10-week period.

One positive feature of our proposed approach is the potential shift of locus of control of “reminders to use the arm” away from the parent and towards the young person *via* the devices and application. This is an appropriate shift for older children, and parents are generally supportive of this (49). The proposed approach would not restrict independence, in contrast to the short-term effect of interventions such as constraint therapy. Our system also has the potential to shift the emphasis away from therapy sessions and more towards increasing arm use in naturalistic settings.

### 4.4. “Technology-enhanced rehabilitation” taxonomy for the upper limb

A recent systematic review of interactive wearable systems for upper body rehabilitation led to a helpful taxonomy comprising *sensing technology*, *feedback modalities* and *system measurements* (39). Regarding *sensing technology*, accelerometers are one of the most used sensors in interactive wearable systems for the upper limb (39). Interpretation of the output requires careful consideration. Acceleration detected in a device worn at the wrist could occur for numerous reasons. We plan for this reason to use two wrist-worn devices and compare the ratio of activity at each wrist. Other possible approaches (which require more detailed computation) include automated recognition of specific patterns of signals indicating the type of activity being undertaken. A simple example of this is provided by Howcroft et al., specifically studying wrist extensions (50), using a bespoke device strapped to the forearm in addition to a “wearable” glove. Machine learning of movement types is possible and may allow for very detailed analysis of data in future. However, approaches such as this require a high number of participants and many time-consuming annotations.

With respect to *feedback*, we intend to provide a brief vibratory stimulus to the affected wrist through the device on an up to hourly basis as a prompt to move, if the level of activity in the preceding hour falls below a threshold set using the baseline data. We will also provide summary feedback data through the smartphone application. Both forms of feedback have been used effectively in other studies (39).

With respect to *system measurements*, our approach differs from most, in measuring “amount of use” of the affected upper limb relative to the unaffected limb: this is helpful in identifying potential improvement but has the disadvantage of requiring two wristbands to be worn. In healthy adults, there is little difference between the amount of use of the two upper extremities (51). Long-term after stroke, adults continue to move the affected arm about half as much as the less-affected arm. The ratio of movement measured with accelerometry reflects real-world arm use (52). Likewise, in typically developing children, there is no difference between arm swing on each side, whereas children with hemiplegia show reduced arm swing on the affected side (53). Young children (aged 2–6 years) with hemiplegia moved their affected arm around 86% as much as the less-affected side (54). Importantly, whilst their accelerometry data correlated with laboratory measurements of motor capacity, it did not correlate with parent-reported real-world arm use. However, it remains possible that arm movements in older children with hemiplegia more closely reflect the pattern seen in adults with stroke: data from Beani et al. (24) are in support of this.

### 4.5. Limitations

Study limitations were largely around recruitment. We were unable to recruit SENCOs to attend the workshops; this likely reflects their many other time commitments but was frustrating given the key role of the school environment to the proposed study. Through remote contact we were able to obtain the opinions of two SENCOs. Recruitment of young people with hemiplegia and their families through social media was also challenging. The proof-of-concept study will recruit through clinicians, which is likely to be effective based on our past experience (55). Furthermore, no fathers of children with hemiplegia were able to attend, and only one participant with hemiplegia was female. Given that there is a gender bias in preference for different types of computer game, it would have been preferable to have had a more even gender split.

## 4. Conclusion

In conclusion our participatory design workshops, engagement with the young persons' advisory group process and consultation with SENCOs were instrumental in informing our plans for the proof-of-concept study.

## Data Availability

The datasets presented in this article are not readily available because; The datasets generated for this study are not publicly available in the interests of patient confidentiality. Requests to access the datasets should be directed to; anna.basu@newcastle.ac.uk.
