# Supplementary material for: Wrist-Worn devices to encourage affected upper limb movement in unilateral cerebral palsy: Participatory design workshops
Source: Front Rehabil Sci. 2022 Dec 21;3:1021760. doi: 10.3389/fresc.2022.1021760 (PMC9812553; doi:10.3389/fresc.2022.1021760)
Supplement: Supplementary file 1 [file Datasheet1.pdf]

Development of 'wearable tech' for hand and arm rehabilitation topic guide

The focus of the workshops is to enhance development of the app and the wrist-worn device through prototype evaluation to guide iterative design change. This is a general guide to the workshops.

Things to bring

- Refreshments
- Information sheets
- Consent/assent forms
- Data collection sheets
- Prototype devices
- Name cards and marker pens
- Pens (biro and coloured)
- A3 sheets of paper
- Post-it notes
- Smartphone
- Blank school timetable

Plan of the workshops

- Introduction (5mins)
- Introduction of all group members (5mins)
- Overview of the study aims and approach (10mins)
- Prototype evaluation (40mins)
- Discussion and feedback (30mins)

Location

- Open Lab

- **Ensure informed written consent/assent obtained from all before proceeding**. **Data capture forms: collect basic demographic information.**

Introduction

- Explain the purpose of the study – to help increase arm activity through the use of wrist-worn device and an app.
- Purpose of the workshop – iterative design changes to the prototype, discussions as to existing knowledge of technology, competition vs comparison from own performance and goals and design of the user interface.
- Explain that we need their views and feedback to do this.
- Remind participants this will be audio recorded.
- Expected duration of the workshop sessions, explain that the participants do not have to attend every workshop though they can attend more than one.
- Lay ground rules – make the participants aware that there are no right and wrong answers, there may be disagreements and discussions, and there is a need to contribute in order to gain all opinions which we value, however if they don't want to contribute they don't have to. One person speaking at any time and to respect confidentiality.

Start audio recording

Introductions of the research team and the rest of the group

Share information about the prototype to date

Example discussion points

- Use of social media and games by young people – what works well and why
- Devices, apps and school
- Capturing and sharing data
- Teenagers and therapy – taking ownership
- What would you change with the device and app? How would you change it? How will it help and be useful? What problems or pitfalls might need to be overcome?

Feedback

- Thank the participants
- Are we asking the right questions?
- Do the participants feel like they have got the most of the workshops and had a chance to contribute as they had hoped?
- Any final questions

Conclusion

- Thank the participants for their input and ideas
- Reinforce what the participant's information will be used for, and reaffirm confidentiality
- Remind participants if they would like they can attend more than one workshop.
